# Bat mating systems—A review and recategorisation

**DOI:** 10.1002/ece3.70149

**Published:** 2024-08-15

**Authors:** Annabel Dorrestein, David Westcott, John M. Martin, David Phalen, Karrie Rose, Justin A. Welbergen

**Affiliations:** ^1^ The Hawkesbury Institute for the Environment Western Sydney University Richmond New South Wales Australia; ^2^ Commonwealth Scientific and Industrial Research Organisation, Land and Water Atherton Queensland Australia; ^3^ Sydney School of Veterinary Science, Faculty of Science University of Sydney Sydney New South Wales Australia; ^4^ Australian Registry of Wildlife Health Taronga Conservation Society Australia Sydney New South Wales Australia

**Keywords:** bats, lek, mammals, mating system, monogamy, polygyny, social organisation

## Abstract

Mating systems, influenced by the social and ecological environment and individual attributes, are fundamental components of animal social organisation, impacting behaviour, animal distribution, ecosystem processes, individual reproductive success, and population dynamics. Bats are of particular interest for studies of mating systems as they are thought to exhibit a greater diversity in mating systems than any other mammalian order, and thus make great models for improving our fundamental understanding of causes and consequences of social organisation. Here, we review the current knowledge of bat mating systems. Our analyses show that research on bat mating systems has not kept pace with research on bats in general and that traditional typologies do not accommodate the mating system of several species. Therefore, we propose an alternative, functional framework to categorise mating systems of bats and by extension of other taxa. We argue that mating systems can be classified according to a male reproductive skew continuum, with an increasing skew from monogamy to true lekking. We include an additional category of lek‐like mating system along the continuum to account for previous trans‐categorical cases that have the appearance of resource defence but are functionally akin to a lek. The new framework has a total of seven categories: promiscuity, monogamy, female defence polygyny, resource defence polygyny, a lek‐like mating system, exploded classical lek, and clustered classical lek. Applying this framework to bats reveals that lek mating systems are more prevalent in bats than previously recognised. It is our aim that this review and the proposed framework provide a greater understanding of bat mating systems particularly and provoke research into the factors that shape mating systems across animal taxa more generally.

## Graphical abstract

Mating systems play crucial roles in behaviour, distribution, reproductive success, and population dynamics. Bats, known for their diverse mating systems, offer valuable insights into understanding the causes and effects of social organisation, yet research in this area lags behind broader bat studies. To address this gap and provoke further research, a new functional framework is proposed to classify bat mating systems, revealing a higher prevalence of lek mating systems than previously acknowledged.

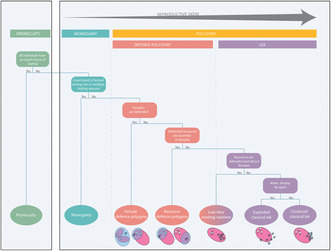

## 1 INTRODUCTION

Social organisation describes the patterns of interactions between individuals within animal societies. These patterns are the result of the choices that individuals make when assessing reproduction and survival options. Social organisation is a topic of fundamental behavioural ecological interest because it provides insights into the structures of animal societies. Mating systems are the social interactions in the specific context of reproduction and result from the decisions individuals make to maximise their reproductive success and from the ensuing conflicts that arise when individuals compete for access to mates and the resources necessary for reproduction (Bradbury & Vehrencamp, 1977; Davies et al., 2012; Emlen & Oring, 1977). Thus, mating systems are the result of individuals maximising their fitness and as such are a crucial component of social organisation.

Historically, mammalian mating systems have been categorised based on the presence of male parental care, the defensibility of the female range, and the stability and size of female groups (Clutton‐Brock, 1989; Emlen & Oring, 1977). In his review, Clutton‐Brock (1989) divided mammalian mating systems into categories ranging from obligate monogamy to leks and roving males, many of which are associated with a variety of forms of mate guarding, such as defence of females, or defence of feeding and mating territories. Following these categorisations, the most prevalent mammalian mating system is ‘resource defence polygyny’ (Clutton‐Brock, 1989).

Bats (Chiroptera) make great models for improving our fundamental understanding of causes and consequences of mating systems. Many species of bats are highly gregarious with tremendously varied ecological roles and life histories (Barclay & Harder, 2003; Racey & Entwistle, 2000). Consequently, bats exhibit a wide variety of mating systems, ranging from monogamy to promiscuity and the gamut of mating systems in between (McCracken & Wilkinson, 2000). In their review of bat mating systems, McCracken and Wilkinson (2000) based their categorisation on characteristics of the female group, that is, seasonality, stability, and composition. However, this scheme focused on the social outcomes of interactions between individuals rather than on the functional foundations that lead to those outcomes, that is, the underlying social and ecological selective pressures that shape individual decisions (Clutton‐Brock, 1989). Furthermore, in the decades since the seminal reviews by Clutton‐Brock (1989) and by McCracken and Wilkinson (2000), great strides have been made in the study of bat mating systems, due to inclusion of a broader range of taxa, improvements in phylogenetic information, and the application of genetic analyses to the study of parentage (Burland et al., 2001; Dechmann & Kerth, 2008; Heckel & Von Helversen, 2003; Hua et al., 2013). Moreover, the integration of genetic studies into mating system research has unveiled discrepancies between social and genetic relationships among mates and their offspring, leading to a delineation between ‘genetic mating systems’ and ‘social mating systems’ (Gowaty, 1996). Additionally, it is becoming increasingly accepted that mating systems exist along a continuum, as a growing number of mating systems appear to fall in between existing categories, or are trans‐categorical (Alonso et al., 2012; Emlen & Oring, 1977; Kotrschal & Taborsky, 2010). For example, species that exhibit mating systems that lie between resource defence polygyny and lek mating systems, neither description adequately captures the underlying selective pressures responsible for this apparent hybrid mating system (Alonso et al., 2012; Welbergen, 2005). These examples suggest that current strict categorical classifications are not covering the wide range of observed mating systems.

Mating systems are fundamentally important in shaping ecosystem processes (Karubian et al., 2012; Westcott et al., 2005) and disease dynamics (Christley et al., 2005). Furthermore, mating systems affect the size of the effective breeding population (*Ne*) and thus may contribute to inbreeding depression (Caro & Eadie, 1998; McEachern et al., 2009; Sutherland, 1998), and for such reasons knowledge of mating systems is also important for conservation management. For example, knowledge of a species' mating system informs effective breeding programmes (Sutherland, 1998) and helps identify important habitat for conservation (Cotterill & Fergusson, 1999; Parsons & Jones, 2003). Hence, the study of mating systems is imperative for both fundamental and applied science.

In this review, we first describe recent progress in the study of bat mating systems. We argue that the mating systems of many species now need recategorisation, and the existing mating system categories are inadequate for accommodating all cases. We then propose a novel functional framework for categorising mating systems in bats, building on Clutton‐Brock's (1989) framework by incorporating male reproductive skew as the central organising principle. Finally, we reclassify all known bat mating systems according to this new framework and identify promising new areas for research.

## 2 RECENT PROGRESS

While a recent review summarised the progress of bat reproductive biology (Ocampo‐González et al., 2021), the last comprehensive review focussed on bat mating systems was done by McCracken and Wilkinson (2000), and summarised the known mating systems of 66 bat species. Since then, the mating systems of 18 additional bat species have been described, bringing the total to 84 (5.7%) of the 1469 extant species (Simmons & Cirranello, 2023; Table 1; Appendix A). While this equates to an approximate 27% increase in the number of species for which mating systems have been described, due to taxonomic revisions and discoveries of new species, this in fact represents a 1.2% decrease in the percentage of Chiropteran species for which the mating systems have been described since McCracken and Wilkinson (2000). Despite this overall proportional decline in the mating systems described, additional mating system descriptions are now available for species in the Molossidae, Mystacinidae, Nycteridae, Phyllostomidea, Pteropodidae, and Vespertilionidae (Table 1). These new mating system descriptions represent species with diverse life histories across the Chiropteran phylogenetic tree; however, across all taxa, the mating system of most species remains unstudied (Table 1).

**TABLE 1:** The number of bat species per family of which the mating systems had been studied in 2000, how many have been studied to date, and the percentage of species studied per family.

Family	Total bat species	Number of species with known mating system in 2000	Number of species with known mating system in 2023	Percentage of bat species with known mating system in 2023
Emballonuridae	55	7	7	12.7
Hipposideridae	92	2	2	2.2
Megadermatidae	6	2	2	33.3
Miniopteridae	41	2	2	4.9
Molossidae	133	4	6	4.5
Mystacinidae	2	0	1	50
Noctilionidae	2	1	1	50
Nycteridae	14	3	3	21.4
Phyllostomidae	226	11	16	7.1
Pteropodidae	202	16	19	9.4
Rhinolophidae	112	3	3	2.7
Vespertilionidae	528	15	22	4.2
All others	56	0	0	0
Total	1469	66	84	5.9

*Note*: Data on recognised bat species come from the online global bat taxonomy database (Simmons & Cirranello, 2023).

**FIGURE 1:**
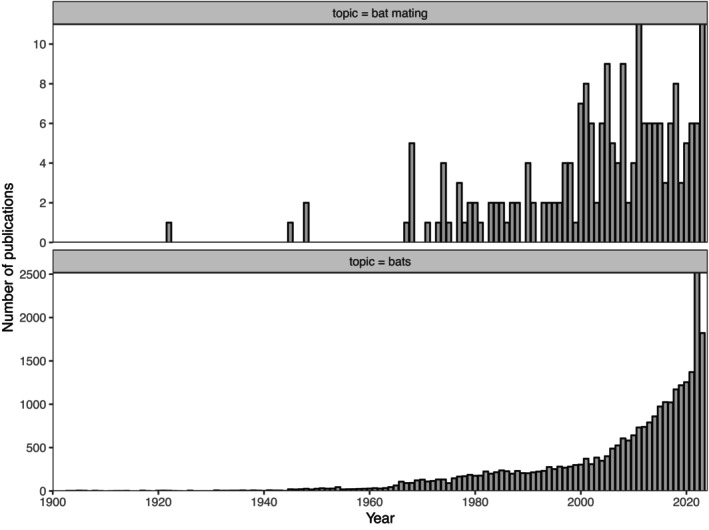
The number of publications on bats and bat mating. Web of Science search on 2 January 2024. Search criteria are described in the text. For publications on bats in general, the following criteria were used: Topic = (bat OR bats OR chiropter* OR flying fox*). Indexes = SCI‐EXPANDED, SSCI, A&HCI, CPCI‐S, CPCI‐SSH, BKCI‐S, BKCI‐SSH, ESCI, CCR‐EXPANDED, IC Timespan = 1900–2023. For publication on bat mating, the following search criteria were used: Topic = (bat AND mating) OR Topic = (bats AND mating) OR Topic = (chiropter* AND mating) OR Topic = (bat AND breeding) OR Topic = (bats AND breeding) OR Topic = (chiropter* AND breeding) OR Topic = (flying fox* AND mating) OR Topic = (flying fox* AND polygyn*) OR Topic = (flying fox* AND lek) OR Topic = (flying fox* AND monogam*) OR Topic = (flying fox* AND promisc*) OR Topic = (chiropter* AND polygyn*) OR Topic = (chiropter* AND lek) OR Topic = (chiropter* AND monogam*) OR Topic = (chiropter* AND promisc*) OR Topic = (bat AND polygyn*) OR Topic = (bat AND lek) OR Topic = (bat AND monogam*) OR Topic = (bat AND promisc*) OR Topic = (bats AND polygyn*) OR Topic = (bats AND lek) OR Topic = (bats AND monogam*) OR Topic = (bats AND promisc*). Indexes = SCI‐EXPANDED, SSCI, A&HCI, CPCI‐S, CPCI‐SSH, BKCI‐S, BKCI‐SSH, ESCI, CCR‐EXPANDED, IC Timespan = 1900–2023.

Although the description of an additional 18 bat mating systems is encouraging, we asked how the relative research intensity into bat mating compared with all bat research. We conducted an exhaustive search of the literature for publications from 1900 to 2020 (inclusive) on Web of Science using the Boolean keywords ‘Chiroptera’, ‘Bats’, or ‘Flying fox’ and compared this to a search of the literature with the combination of Boolean keywords ‘Chiroptera’, ‘Bats’, or ‘Flying fox’, and ‘Mating’, ‘Breeding’, ‘Polygyny’, ‘Promiscuity’, ‘Monogamy’, or ‘Lek’. A generalised linear model for publications per year revealed a significant difference in slope of the number of publications per year, per topic (*F*_3,160_ = 34.085, *p* < .001; Table 2), meaning that research on bat mating systems has not kept pace with research on bats in general (Figure 1). Evidently, research effort into bat mating systems has been lagging, and publications on the topic have, in fact, decreased relative to publications on bats in general since 2000 (Figure 1), and to date, the mating system of 94% percent of bat species remains unknown.

**TABLE 2:** Results of a generalised linear model constructed using the lme4 package in R with number of publications in a year as a dependent variable, publication year, publication topic (two levels: Bat mating research and bat research), and an interaction between year and publication topic as covariables.

	Estimate	Standard error	*t*‐value	*P*
Intercept	−155.4	3068	−0.051	.960
Year	0.080	1.538	0.052	.959
Publication topic	−15,870	3302	−4.806	>.001
Year × publication topic	8.198	1.659	4.940	>.001

## 3 A GENERAL FRAMEWORK FOR MAMMALIAN MATING SYSTEMS

There is much ambiguity around the traditional categories of monogamy, polygamy, and promiscuity, and it has become apparent that mating systems are more varied than previously thought (Jahelková & Horáček, 2011; Jiguet et al., 2000; Toth et al., 2015; Welbergen, 2005). An increasing number of described mating systems do not fit neatly into a single category, but rather fall in between, or are trans‐categorical by exhibiting a combination of characteristics from different mating systems. Varieties of lek mating systems that may lie between resource defence polygyny and classical lek mating systems have been suggested (Alonso et al., 2012; Welbergen, 2005). In addition, it is increasingly recognised that species may exhibit intraspecific variation in mating systems, with species or individuals exhibiting different mating systems depending on the time of day (Günther et al., 2016), time of year (Buzatto & Machado, 2008), or based on factors such as individual condition and status. Consequently, a variety of mating strategies may be shown within a population or species (Brockmann, 2001; Gross, 1996; Gursky‐Doyen, 2010; Isvaran, 2005; Shuster, 2010). To account for such ambiguities, a novel functional framework of mating systems is needed.

Mating systems are primarily determined by resource distribution (Davies et al., 2012). Females mainly maximise their reproductive success by accessing preferred resources, and female dispersion is thus primarily determined by resource distribution (Davies et al., 2012). Males, on the other hand, primarily maximise their reproductive success by defending females or the resources that females need. Depending on how resources and females are dispersed, males can monopolise access to one or multiple females. However, when males are unable to defend economically either females or resources required by females, leks could occur (Bradbury, 1977a; Emlen & Oring, 1977). Leks are aggregations of males each defending a mating territory containing no resources, where they advertise themselves to the females (Bradbury, 1977a). Ultimately, resource distribution affects female dispersion, which in turn affects male distribution; therefore, resource distribution is an important determinant of the array of mating systems that can be observed in nature (Davies et al., 2012).

The variety of mating systems can be characterised by differences in male reproductive success. In species with a 1:1 adult sex ratio, male and female reproductive success is equal on average, but as males can monopolise access to females, there is a higher variance in male reproductive success (see also Bateman's principle; Bateman, 1948). In monogamous species, males have a relatively low variance in reproductive success as, by definition, males tend to be paired up with single female so that males tend to share equally with other males in reproduction; whereas at the other end of the spectrum, males in lek mating systems have a much higher variance in reproductive success (Brown et al., 2009; Clutton‐Brock, 1989), as a few dominant males enjoy a disproportionally large share of reproduction (Mackenzie et al., 1995). Mating systems can thus be placed along a reproductive skew continuum, with an increasing skew from monogamy to lekking. Within monogamy, such a continuum would account for varying degrees of extra‐pair copulations; true monogamy would be on the far end of the continuum, and with an increase of extra‐pair copulations, reproductive skew increases. When the polygyny threshold is reached, females pair with an already‐paired male, forming a harem (i.e., a group of multiple females with one or two males), leading to a polygynous mating system. Similar to monogamy, polygyny has been described as having varying degrees of male reproductive skew and can also be placed along the proposed continuum. Likewise, a continuum has been proposed between resource defence polygyny and lek mating systems (Alonso et al., 2012; Kotrschal & Taborsky, 2010). Here, we suggest that all known mating systems, from ‘true’ monogamy to ‘true’ lekking, can be placed along a male reproductive skew continuum. This continuum would account for trans‐categorical mating systems and would be able to accommodate varying degrees of monogamy and polygyny.

**FIGURE 2:**
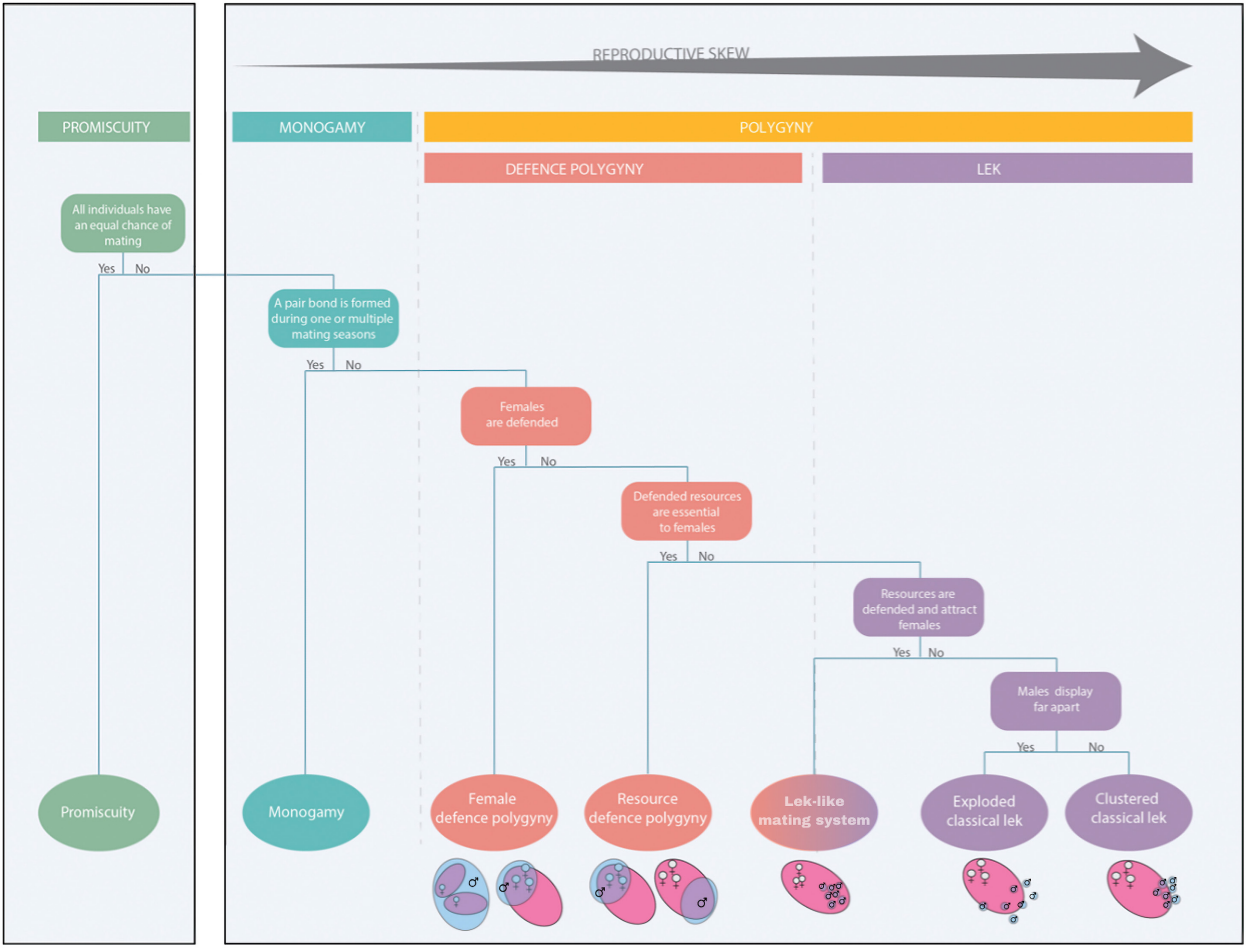
A classification of mating systems in mammals. Terms are defined in the text and in Table 3. The pink ovals represent home range of one or multiple females (pink), as depicted by the female symbols, and every blue oval represents the area defended by a single male, i.e., a mating territory (blue). The figure represents a schematic representation of the positioning of male mating territories in relation to the female home range per mating system. The lek‐like mating system is a new proposed mating system.

In this review, we re‐evaluate the existing mating system categories to account for new information and clarify the relationships and the diversity in mating systems observed in bats. We retain the overarching categories ‘promiscuity’, ‘monogamy’, and ‘polygamy’, but we provide more detailed sub‐categorisation within these groups, based on the underlying social and ecological factors, organised along the skew continuum (Figure 2). The different mating systems and their characteristics are discussed in‐depth.

### 3.1 Promiscuity

The term promiscuity is sometimes used interchangeably with polygynandry. The two mating systems share some characteristics; they are both multi‐male multi‐female mating systems. However, contrary to polygynandry, promiscuity is considered indiscriminate, random mating (Garcia‐Gonzalez, 2017), whereas polygynandry occurs within a social structure, so that mating is non‐random. Promiscuity would be characterised by minimal male reproductive skew, as individuals mate randomly and thus have an equal chance of mating. Under this definition, promiscuity is relatively rare, as it is beneficial to one sex (usually presumed to be females due to their higher investment in individual gametes and offspring) to exhibit mate choice (Darwin, 1871). Promiscuity occurs when female ranges are too large to defend and female groups are unstable in composition (Clutton‐Brock, 1989), making it unfeasible for males to defend females or resources, or to set up mating territories.

### 3.2 Monogamy

Monogamy is defined as a mating system whereby individuals of both sexes only have one mate (Kleiman, 1977; Moller, 2003; Reichard, 2016; Table 3). The male and female often form a pair bond, which is ‘a close relationship through courtship and sexual activity with one other animal’ (Simpson et al., 1989), and the pair remains together through one or multiple breeding seasons (Kleiman, 1977). However, as more genetic studies are conducted, it is clear that most socially monogamous animals are not genetically monogamous and participate, sometimes frequently, in extra‐pair copulations (Shuster & Wade, 2003). This phenomenon has been observed in many ‘monogamous’ mammals (Fietz et al., 2000; Goossens et al., 1998; Reichard, 1995), leading to the following three recognised types of monogamous relationships: (i) social monogamy, pairs have extra‐pair copulations; (ii) mating monogamy, having a single sexual partner; and (iii) reproductive monogamy, exclusive and ongoing contributions of two individuals to offspring (Reichard, 2016). Reproductive monogamy will not be included in this review, as it mostly occurs in eusocial insects, where the queen stores sperm of a single male for all her offspring, and this is not known to occur in bats. In this review, we consider bat species that form pair bonds, regardless of extra‐pair copulations, to be monogamous. Social monogamy and mating monogamy are thus both included in the general monogamy category (Figure 2; Table 3).

**TABLE 3:** Proposed redefinition of mating systems.

Mating system	Description	Bat species examples
Promiscuity	Mating system where mating occurs randomly among males and females, but reproductive success may vary between males, due to sperm competition	Little brown bat (*Myotis lucifugus*; Thomas et al., 1979; Wai‐Ping & Fenton, 1988; Watt & Fenton, 1995)
Monogamy	Mating system where a single male and single female either mate exclusively with each other or form a pair bond for one or multiple seasons	
Mating monogamy	A single male and a single female form a pair bond and mate exclusively with each other during a mating season. They do not necessarily maintain this pair bond during multiple mating seasons. As no extra‐pair copulation occurs by definition, this can be regarded ‘true’ monogamy	Uncertain
Social monogamy	A single male and single female form a pair bond but may participate in extra‐pair copulations. A pair may stay together for one or multiple mating seasons	Painted woolly bat (*Kerivoula picta*; Bradbury & Vehrencamp, 1977; Brosset, 1962; Funakoshi, Fukui, Yamamoto, Mizuno, Osawa, Osawa, Yoshikura, Minesita, Sato, Tsuji, Matsumura, et al., 2015)
Polygamy	Mating system where one or both sexes mate with multiple partners	
Female defence polygyny	Males defend multiple females within the females' home ranges to the exclusion of other males. When the females move away, the male moves with the group. Females may secure extra‐group/harem copulations. Sometimes multiple males defend the females	Bonin flying fox (*Pteropus pselaphon*; Sugita & Ueda, 2013)
Resource defence polygyny	Males set up a mating territory in or around a resource (roost or foraging site) that is essential to females. They mate with and may defend the females that are using this site. Females may participate in extra‐group/harem copulations, or reside in multiple harems over the course of a mating season. In some instances, multiple males defend a mating territory	Greater sac‐winged bat (*Saccopteryx bilineata*; Bradbury & Vehrencamp, 1976)
Lek‐like mating system	Males establish mating territories that include resources that are not essential to females but that act as a proxy of male quality. Mating territories are clustered as in a lek. Females can move freely among males	Grey‐headed flying fox (*Pteropus poliocephalus*; Welbergen, 2005)
Exploded classical lek	A male defends a mating territory that does not contain resources that are essential to females. Females base their mate choice solely on male quality. Mating territories are spaced out at the scale of the lek but clustered at the landscape scale	Possibly: Franquet's epauletted fruit bat (*Epomops franqueti*; Bradbury, 2016; Toth & Parsons, 2013)
Clustered classical lek	A male defends a mating territory that does not contain resources that are essential to females. Females base their mate choice solely on male quality or phenotypic proxies thereof. Mating territories are highly clustered in space	Hammer‐headed bat (*Hypsignathus monstrosus*; Bradbury, 1977a, 1977b)

Monogamy can occur when resources are distributed such that females are solitary or range too widely for a male to defend multiple females, or abundant resources prevent effective defence of more than one female (Davies et al., 2012). Forming a monogamous pair with a female can help ensure mating access to this female and hence his paternity to her offspring. To the female, monogamy can provide direct benefits, such as when male assistance in rearing is required to ensure survival of the offspring (Clutton‐Brock, 1989), either by improving resource provisioning to offspring, or by preventing sexually selected male infanticide (Reichard, 2016).

### 3.3 Polygamy

Polygamy is defined as a mating system whereby one or both sexes have access to multiple mates, and mating occurs nonrandomly (Table 3). Polygyny is defined as males having access to multiple mates and polyandry as females having access to multiple mates. In more than 90% of mammal species, a single‐male mates with multiple females, and polygyny is thus thought to be the most common mating system in this taxon (Clutton‐Brock, 1989; Kleiman, 1977).

Females of many socially polygynous mammal species engage in genetic polyandry by participating in extra‐harem copulation or switching harems to mate with higher quality males, avoid infanticide by concealing paternity (Boulton & Shuker, 2013; Reichard, 1995), or prevent mating with male descendants (Nagy et al., 2007). However, social polyandry is rare in mammals. Due to gestation and lactation, female reproductive success is constrained by access to resources rather than mates, resulting in limited male parental care (Safari & Goymann, 2021) and rare sex‐role reversal (Eens & Pinxten, 2000; but see Bro‐Jørgensen, 2007). Females, investing heavily in offspring survival, are selective in mate choice (Orians, 1969) and may join already‐paired males if they provide better genes or resources (Pribil, 2000). This decision involves a trade‐off, as females must share territory and mating access with other females, making it beneficial only if the value of joining outweighs these costs, a concept known as the polygyny threshold (Orians, 1969; Wittenberger, 1981). In polygynous systems, pair bonds can coexist with harem structures, and females may pair monogamously with lower‐quality, unmated males if sharing resources and mating attempts with others is too costly (Markus, 2002; Nelson, 1965; Welbergen, 2005). Polygyny implies males' potential to monopolise multiple females, but monogamous pairings can also occur (Garnier & Schradin, 2019). Males adopt various strategies to monopolise mating access based on the distribution of females, which will be discussed below.

#### 3.3.1 Female defence polygyny

Female defence polygyny is defined in this review as a male defending multiple females within the female's home range to the exclusion of other males (Figure 2, Table 3, Clutton‐Brock, 1989). This mating system occurs when multiple solitary females can be defended, or when a female group has a stable composition, and their range is defendable, as it allows males to compete for and defend access to such female groups (Davies et al., 2012). There are two different types of female defence polygyny: female range defence and female group defence. Female range defence occurs when males are able to defend the home ranges of multiple solitary females.

Within female group defence, a group of females can be defended by a single male or by multiple males (Table 3). When female groups are large, multiple males sometimes defend the territory together. These males are often relatives. Such joint defence of the females is often necessary for economic defence of larger group of females but may also lead to an increase in tenure (Bygott et al., 1979; Davies et al., 2012).

#### 3.3.2 Resource defence polygyny

Resource defence polygyny is defined in this study as males defending resources that attract or are critical for females to monopolise mating access to females. Females obtain access to essential resources by mating with these males (Figure 2; Table 3). This mating system tends to occur when females cannot be defended, for example, when female groups have an unstable composition or when intruder pressure is too great. There are two general types of resources critical to female fitness, foraging and roosting resources, and males can defend either or both.

Resource defence polygyny is usually described as a harem‐based form of polygyny, but multi‐male forms occasionally occur (see Keeley & Keeley, 2004; Rodríguez‐Herrera et al., 2019). This does not mean, however, that females in a harem mate only with the male(s) in their harem. When female movements are not restricted by males, and resources are abundant and similar in quality, there is no benefit of staying in a particular place and females could take the opportunity to mate with multiple mates (Campbell et al., 2006; Nagarajan‐Radha et al., 2024).

Female defence polygyny and resource defence polygyny are both widely recognised mating systems. However, they do not appear to be clearly distinct categories, but rather different parts of the same continuum. Sometimes males defend both resources and females, or shift between them, and thus species will not always fit neatly into a single category (Buzatto & Machado, 2008; Ostfeld, 1987).

#### 3.3.3 Lek mating systems

When female groups are even more unstable or the female ranges larger still (e.g., species moving large distances during seasonal migration), it becomes unfeasible for males to defend resources or females to monopolise reproduction. Males can then set up mating territories containing no essential resources, or no resources at all, and attract females by competitive display and courtship rituals, usually auditory or visually, which is called a lek mating system (Bradbury, 1981; Clutton‐Brock et al., 1993; Höglund & Alatalo, 1995; Figure 2). Males aggregate at this ‘lek’, that is, the communal display area, and set up mating territories for the sole purpose of attracting females. Four criteria have been defined for lek mating systems (Bradbury, 1981; Bradbury & Gibson, 1983): (1) males aggregate for display, (2) females can select their males, (3) males do not provide resources, including paternal care, and (4) the only benefit females gain is access to males for reproduction. We followed the same criteria but excluded paternal care from the flow chart and table, as there has been no evidence of male parental care in bats to date (but see Hosken & Kunz, 2009).

##### Classical leks

All of Bradbury's criteria (1981) are unambiguously characteristic of classical leks. While classical leks sometimes contain resources, these do not serve to attract females and may be incidental and possibly provide some resources for the male (Jiguet et al., 2000). Within leks, two subcategories can be defined: the classical lek and the exploded classical lek. The classical lek is characterised by spatial aggregations of male mating territories that are tightly clustered, in many cases sharing boundaries (Lill, 1974; Robertson et al., 2008). In an exploded lek the degree of clustering is reduced, and male display territories are separated by unoccupied habitat, albeit there is no formal distinction between classical and exploded leks other than their degree of spatial clustering.

Resource defence polygyny and lek mating systems have been argued to be the two ends of a continuum of the relative importance of resources in a mating territory. In resource defence polygyny, the defended resources attract females, while in a lek mating system male phenotypic characteristics facilitate female choice. However, using path analysis to establish the relative importance of phenotypic characteristics and food resources, Alonso et al. (2012) suggested that there is a gradation in the significance of both resources and male traits in female choice. While such a continuum covers a great part of the observed variation in polygamous mating systems, it does not accommodate situations where females use the defended resources as an indicator of male quality, rather than the resources giving direct benefits to females. Here, resources comprise a males' extended phenotype, and as such signal male quality in terms of its ability to command these resources. Using observed bat mating systems, we suggest the need for recognising this transitional state by the inclusion of the lek‐like mating system.

##### Lek‐like mating systems

As previously discussed, there are likely mixed mating systems occurring between resource defence and lek mating on the continuum. One such mating system may occur when resources are not essential but are used by females to assess male quality. This mechanism has been suggested to occur in the grey‐headed flying fox (*Pteropus poliocephalus*; Welbergen, 2005) but may apply more broadly. Individuals of this species aggregate year‐round, but during the mating season males compete for mating territories in the centre of the roost, and the more central males were heavier, larger, and in better body condition than the peripheral males (Welbergen, 2005). Subsequent to the formation of mating territories, females predominantly associate with central, higher quality males. As males defend locations used for roosting, and females make use of this resource, it could be argued that this mating system fits the criteria for resource defence polygyny. However, roosting branches are widely available outside of these mating territories and are thus not considered a limiting (or ‘essential’) resource. Additionally, females occupied a roosting position before moving into mating territories, and they moved freely between different mating territories (Welbergen, 2005). This suggests that they did not gain any direct benefits from staying with a central male, such as a decrease in exposure to predation; nor did females seem to gain protection from harassment by subordinate males as females in more central locations in fact appear to be harassed more often (Welbergen pers. obs.). Thus, in the grey‐headed flying fox, it appears that the locale in the roost provides an honest indicator of male quality, rather than providing direct benefits to females (Welbergen, 2005). This type of behaviour is not exclusive to bats; it has been described in cichlid fish, where males defend patches of homogenously distributed food resources, visited by females, who sample several territories (Kotrschal & Taborsky, 2010).

To accommodate a mating system where males defend a non‐essential resource that females use to assess male quality, we introduce this third, previously unrecognised, lek mating system: the lek‐like mating system. This mating system has the appearance of a resource defence polygyny but is functionally akin to a lek, as females are not dependent on the defended resource. The lek‐like mating system unequivocally complies with three of Bradbury's criteria for leks: (1) males aggregate within a resource, (2) females select their males based on the quality of their resource, and (3) males do not provide parental care. We argue that the fourth criterion, the only resource females find at these sites are the males themselves, is also met, as by Bradbury's criteria a lek is not distinguished by the absence of resources but by whether males control access to resources to obtain mating opportunities (Bradbury, 1981; Bradbury & Gibson, 1983).

##### Hidden lek

The hidden lek is a form of lek that has hitherto not been recorded in mammals. This lek occurs in monogamous or polygynous birds that participate in extra‐pair copulations. For these extra‐pair copulations, males establish their mating territories that are either separate or the same as their roosting territories, in leks. These leks provide easy access and choice for females that are receptive to extra‐pair copulations (Wagner, 1998). Males thus adapt their mating strategies to accommodate genetic polyandry.

Five criteria have been proposed for hidden leks: (1) social monogamy or social polygyny, (2) aggregations of territorial males, (3) Females are receptive to EPCs and have opportunities to select extra‐pair mates, (4) no parental care from extra‐pair males, and (5) extra‐pair males do not monopolise resources required by females (Fletcher Jr & Miller, 2006). This mating system has not been described in mammals to date. However, social polygyny does occur in bats, and it is likely the other criteria could also apply to social polygynous bat species. It is thus possible males set up hidden leks, providing an interesting avenue for future research.

## 4 CLASSIFYING BAT MATING SYSTEMS

We classified all bat species for which we could find publications on mating system according to the new functional framework based on the male reproductive skew (Appendix A) and elaborate on the classifications below.

### 4.1 Promiscuity in bats

In bats, mating in swarms is often considered to be random, as these swarms consist of large multi‐male, multi‐female groups in which mate choice is difficult to observe. However, mate choice may still occur, albeit possibly cryptic (Wilkinson & McCracken, 2003). Most swarming species congregate at underground sites where individuals engage in vocal displays, and participate in chasing, providing opportunities for mate choice to operate. Furthermore, genetic studies show that some swarming males have higher reproductive success than others, indicating either non‐random mating or sperm competition (Senior et al., 2005; Watt & Fenton, 1995). This phenomenon could indicate a difference between social and genetic promiscuity, similar to the division in monogamy. If the skew in male reproductive success is a consequence of sperm competition, the mating system could still be socially promiscuous. If there is a form of female mate choice, it would most likely be a form of a lek mating system. As swarming is the primary mating event for some bat species (Kerth & Morf, 2004; Rivers et al., 2005; Veith et al., 2004), but it is unknown what mating system is shown, further research is needed to address whether female choice occurs during swarming.

We attributed promiscuity to nine bat species (Appendix A). A convincing example of a promiscuous mating system is found in the little brown bat (*Myotis lucifugus*). In this species, all males seemed to have equal access to females for mating, and copulation occurred immediately after females contacted males. Further, males forced copulations with torpid females, causing females to have no means of mate choice, and likely causing males to not invest in attracting females, but rather inseminate as many females as possible investing a minimum of energy. Indeed, no competition, site defence or fidelity, or evidence of male self‐advertisement was observed (Thomas et al., 1979). The greater long‐nosed bat (*Leptonycteris nivalis*) is also attributed to a promiscuous mating system, as no signs of territoriality, courtship, or formation of a lek or harem have been detected (Caballero‐Martínez, 2004, in Nassar et al., 2016). We also categorised swarming species with no current evidence of non‐random mating as promiscuous, but as mentioned, further research should be conducted to assess whether mating during swarming is truly promiscuous.

### 4.2 Monogamy in bats

The current study shows that in bats with known mating system, 16 species, representing 19% of described mating systems, have been identified as exhibiting pair bonds (Appendix A). Since the review by McCracken and Wilkinson (2000), pair‐bonding has not been reported for additional bat species. However, additional research has been conducted on the painted woolly bat (*Kerivoula picta*) and the Samoan flying fox (*P*. *samoensis*). The painted woolly bat was found to roost in families, numbering three individuals (adult male and female, and young), during three months of the year and to retain their reproductive partner over multiple years (Funakoshi, Fukui, Yamamoto, Mizuno, Osawa, Osawa, Yoshikura, Minesita, Sato, Tsuji, Matsumura, et al., 2015). The Samoan flying fox appeared to form pair bonds only during the mating season, with males establishing 2 km^2^ territories defended with dramatic aerial fights (Banack & Grant, 2003; Brooke, 2001). It is unclear whether these pair‐bonding species participate in extra‐pair mating, as no genetic data are available.

### 4.3 Polygamy in bats

#### 4.3.1 Female defence polygyny

Female defence polygyny was attributed to 13 species (Appendix A). Female range defence has not been found in bats to date. However, bats have cryptic, nocturnal lifestyles, making female range defence difficult to detect. Female group defence is easier to detect, as this often manifests in harem structures at roosts and has been identified in multiple species. Female group defence is found in the greater bulldog bat (*Noctilio leporinus*), where female groups stay together longer than the tenure of a male, which can be up to three reproductive periods (Brooke, 1997). Female group defence polygyny is also found in the swarming Daubenton's bat (*M*. *daubentonii*). Swarming behaviour is often thought to facilitate promiscuous mating (Cope & Humphrey, 1977; Rivers et al., 2005; Veith et al., 2004), but a recent study showed that before swarming commences in this species, dominant males roost and mate with females at maternity colonies, which leads to a skewed male mating success (Senior et al., 2005). We, therefore, chose to categorise the mating system used in this species as female defence polygyny.

Female defence polygyny has been reported for two *Pteropus* species. Females of the Bonin flying fox (*P*. *pselaphon*) form clusters in winter for thermoregulatory purposes. This behaviour creates the opportunity for males to monopolise such clusters for mating, defending these harems against other males. This mating system can thus be described as female defence polygyny (Sugita & Ueda, 2013). Similarly, observational data for the black flying fox (*P*. *alecto*) identified males moving into the female's regular roost spaces and establishing territories around groups of females (Markus, 2002), therefore exhibiting female defence polygyny.

#### 4.3.2 Resource defence polygyny

Resource defence polygyny is relatively prevalent in bats and was attributed to 22 species (Appendix A). The greater sac‐winged bat (*Saccopteryx bilineata*) exhibits resource defence polygyny where the male defends an area both at the roost and at the foraging site (Bradbury & Vehrencamp, 1977). Roost defence polygyny, which can refer to smaller areas within a roost (e.g., in a cave) or to the defence of an entire roost site (e.g., defence of a tent built out of a leave), has been shown frequently in bats (Balmori, 2017; Braun de Torrez et al., 2020; Fernandez et al., 2014; Morrison, 1979; Muñoz‐Romo et al., 2008). There are no convincing cases of defence of just the foraging site described in bats to date.

The great fruit‐eating bat (*Artibeus lituratus*) has been described as exhibiting resource defence polygyny (Muñoz‐Romo et al., 2008). Males defended roosts, and the male that regularly had access to the most females occupied a roost that was considered to be of the highest quality, defined as the highest, most structurally stable, and the least disturbed. In the tent‐making bat (*Artibeus watsonia*), access to females appears to be related to roost defence, as no male–male associations were observed at sites with females. The females did not show fidelity towards one roost site, and thus polygynandry has been suggested (Chaverri et al., 2008), similar to *Cynopterus sphinx* (Nagarajan‐Radha et al., 2024). However, our framework describes the functionality of the mating system while not excluding the possibility of female extra‐harem copulations. The tent‐making bat appears to functionally have a resource defence polygyny, with males defending roosts or roost territories. The females may partake in extra‐harem copulations, or might switch roosts regularly, which still fits within our definition of resource defence polygyny.

In the spotted‐winged fruit bat (*Balionycteris maculata*), males defend territories in cavities of caves, mines, termite roosts, and ant nests, resulting in males forming harems within these territories. Females regularly switch harem and are found to be roosting with three different males over the mating season. The roost cavities are thought to be a critical and defendable resource (Hodgkison et al., 2003), and the species is thus thought to show resource defence polygyny. The Honduran white bat (*Ectophylla alba*) was shown to display resource defence polygyny, as the tents they make for roosting are considered to be a limiting resource (Brooke, 1990). These bats were long thought to have a single‐male form of this mating system. However, a recent study found that 75% of the groups had more than one male (Rodríguez‐Herrera et al., 2019), and the species is therefore now considered to exhibit a multi‐male form of resource defence polygyny.

The lesser noctule bat (*Nyctalus leisleri*) forms harems during the mating season, with low stability in female groups. In their study, Dondini and Vergari (2009) observed that the females aggregated around the most competitive males but did not describe what the males would be defending. If the males did not defend an essential resource, this mating system could better be categorised as lek‐like (below).

#### 4.3.3 Lek mating systems

In studies on bat mating systems, it is often unclear whether resources defended by males are ‘essential’ to the females, which is important because the absence of essential resource in a mating territory is one of the four criteria of a classical lek mating system (Bradbury, 1977a). For the purpose of categorising and further analysis of the mating systems, we chose to be conservative and classify mating systems where it is unclear if resources are essential as resource defence polygyny. Following this, a lek mating system was confidently attributed to 14 species of bat (Appendix A).

One of the proposed physiological prerequisites for lek formation is a high mobility, as it allows females to search for male aggregations (Höglund & Alatalo, 1995). In mammals, lek formation has been hypothesised to be constrained by low mobility, compared to birds, limiting it to large, free‐roaming species (Toth & Parsons, 2013). Bats, however, are highly mobile mammals (see Welbergen et al., 2020; Westcott et al., 2020), and therefore lekking may be expected to be more common in bats (Toth & Parsons, 2013). Yet, lek breeding is reportedly very rare in bats, even though bats display the widest range of mating behaviours among mammals (Altringham, 2011).

Since McCracken and Wilkinson's (2000) review, two additional bat species have been attributed to a lek mating system, *Mystacina tuberculata* and *Centurio senex*, bringing the total species with a lek mating system to three. However, a subsequent review on lek mating in bats (Toth & Parsons, 2013) also identified *Epomops franqueti*, *Epomorphus wahlbergi*, *Pipistrellus nathusii*, *Miniopterus minor*, and *Erophylla sezekorni* as showing lek mating characteristics, and suggested that despite current lack of evidence, lek mating could be more prevalent than previously thought, particularly in temperate bats. Indeed, on further investigation, some mating systems that were previously considered resource or female defence polygyny more closely resemble the lek‐like mating system, predominantly because of the lack of *essential* resources in male mating territories. The buffy flower bat (*Erophylla sezekorni*) shows a mating system that meets all the criteria of a classical clustered lek, except that the mating territories are also situated at roost sites and thus could be providing a resource. The males aggregate to establish mating territories where they exhibit wing displays and produce garlic‐scented supraorbital secretions. They hold their mating territory for the entire mating season. Females roost both in the display area and on the periphery, suggesting that roost sites are non‐limiting (Murray & Fleming, 2008). This shows that males aggregate for display and do not provide resources, and females can select their mate. The mating system thus classifies as a lek‐like mating system.

The banana pipistrelle (*Neoromicia nana*, formerly *Pipistrellus nanus*) was previously attributed to a single‐male, multi‐female mating system (McCracken & Wilkinson, 2000). We propose that their mating could resemble a lek‐like system. Males defend ephemeral roost sites, which are not limited to the females, as there were more roost sites than males. Moreover, females do not show fidelity towards males or their roost (Happold & Happold, 1996). Thus, the aggregating males do not provide an essential resource, and the females select their mates. There is no recorded obvious display behaviour, but the positioning of the defended ephemeral roost site could be an indicator of quality to females. As such, the banana pipistrelle is likely to have a lek‐like mating system.

The soprano pipistrelle (*Pipistrellus pygmaeus*) could also be exhibiting a lek‐like mating system. This species is shown to mate away from hibernacula, and the authors suggest that males could be displaying close to the nursery colonies (Bartoničková et al., 2016). Additionally, lactating females were visiting males and roosting with them for a day (Bartoničková et al., 2016). This could be evidence of a lek mating system, albeit unclear which type.

In Spain, male European free‐tailed bats (*Tadarida teniotis*) defend roosts, from where they emit calls to attract females (Balmori, 2017), clearly displaying to attract females. The authors classify this as resource defence polygyny. However, from their study it is not clear whether roost sites are a limiting resource. The males defending a higher roost seem more successful, but, similar to grey‐headed flying foxes, this could be an indicator of male quality (see above; Welbergen, 2005). Every night, males actively display to attract females before their return to the roost sites, which provides a further suggestion that this species exhibits a lek‐like mating system.

The exploded classical lek potentially occurs in Franquet's epauletted fruit bat (*Epomops franqueti*) (Bradbury, 2016; Toth & Parsons, 2013). This species establishes relatively large mating territories, of 100–200 m in diameter, from which they display whistle‐like vocalisations to attract females. The females visit these territories, where they hover in front of the displaying male, and sometimes join in a duet. Copulations, however, were not observed, and female mate choice thus cannot be confirmed (Bradbury, 1981).

Both Peters' epauletted fruit bat (*Epomophorus crypturus*) and the almost similar looking Wahlberg's epauletted fruit bat (*E*. *wahlbergi*) could be attributed to a lek‐like mating system, as the males of these species call from nightly mating territories (Adams & Snode, 2015; Fenton et al., 1985). It is unclear, however, whether males were aggregated, and if so, if it would classify as a clustered or exploded lek, or whether it is a lek at all.

Only three bat species have been confirmed to exhibit a clustered classical lek mating system. The hammer‐headed bat (*Hypsignathus monstrosus*) sets up traditional display areas, from which they vocalise to attract females (Bradbury, 1977a). The males of the lesser short‐tailed bat (*Mystacina tuberculata*) set up clustered singing roosts that were defended by the territory holders and visited by females for mating purposes only (Toth et al., 2015). The third species confirmed to have a clustered classical lek mating system is the wrinkle‐faced bat (*Centurio senex*). Males display from perches in a small area by moving their wingtips and vocalising. When approached by a female, they beat their wings and produce a loud whistling call. The authors observed copulation following such an approach (Rodríguez‐Herrera et al., 2020).

A clustered classical lek has further been suggested for the least long‐fingered bat (*Miniopterus minor*) (Toth & Parsons, 2013). Males of this species aggregate and exhibit display behaviour in a small, eroded hollow. Only the heaviest males acquired a spot in this so‐called mating dome. However, copulation has never been observed, and the classical lek mating system can therefore not be confirmed (McWilliam, 1990). The California leaf‐nosed bat (*Macrotus californicus*) could also be exhibiting a clustered classical lek, as males display in an abandoned mine that is normally not used for roosting. The males display wing flapping and vocalisations to attract females, and the females do not gain any other benefits than access to males (Berry & Brown, 1995). A clustered classical lek has also been suggested for the grey sac‐winged bat (*Balantiopteryx plicata*), as the males aggregate and display by calling during a short copulation period. The females visit these aggregations for the sole purpose of mating (Nagy et al., 2014).

One of the world's best‐studied bat species, the common pipistrelle (*Pipistrellus pipistrellus*), can be attributed to a lek mating system. This species was previously attributed to a harem‐form mating system (McCracken & Wilkinson, 2000), but in 2006 they were observed to set up territories with a diameter of 100–200 m in an urban environment, where they emitted vocalisations. The territories were more densely clustered in the city centre, where the winter colonies were situated, than in the periphery where nursery roosts were situated. This arrangement ensured females would pass through mating territories when assessing winter roosts (Sachteleben & von Helversen, 2006). This mating system could thus be defined as a clustered classical lek. Interestingly, despite being very well‐studied, mating has not been observed, and further study is necessary to confirm the species' mating system.

## 5 DISCUSSION

In this review, we established a functional framework for categorising bat mating systems, based on the male reproductive skew continuum. When assessed against our framework, bat mating systems exhibit enormous variety, ranging from promiscuity to true lek mating systems, and everything in between (Appendix A). The majority of bat species (51%) exhibit a form of polygyny, similar to mammals in general, where a vast majority (>90%) exhibit polygyny (Clutton‐Brock, 1989). However, many bat species that have been described to exhibit resource defence polygyny could potentially be described as having a lek, or a lek‐like mating system, under our classification. We expect that systematic and detailed research into bat species with suspected lek mating systems will reveal more variations within lek mating systems, likely filling the continuum between resource defence and classical lek mating systems. Further research in bat mating systems generally will provide much needed insights into the diversity of mating systems, which is critical to a better understanding of social organisation.

Genetic studies have become a standard part of mating systems research, and it has become clear that the observed social relationships between females and males often do not match the genetic relationships between mates and their offspring. This discrepancy led researchers to differentiate between ‘genetic mating systems’ and ‘social mating systems’ (Gowaty, 1996). Females often undertake extra‐pair, or extra‐group copulations, and genetic polyandry is now considered a common trait, providing females with a variety of direct and indirect reproductive benefits (Boulton & Shuker, 2013; Pizzari & Wedell, 2013). The proposed male reproductive skew continuum aims to integrate genetic and social mating systems. ‘Genetic polyandry’ in monogamy is accounted for by the male reproductive skew, with an increase in extra‐pair copulations resulting in a higher male reproductive skew. The social mating systems can still be considered monogamy. Genetic studies are imperative to determining the position of a mating system on the continuum, whereas the social mating system helps determining the category within the continuum.

Genetic studies are also vital to assessing the mating system of swarming species. Such studies could reveal a non‐random male reproductive skew, indicating sperm competition or female choice. Female choice would indicate a mating system with a societal structure, such as a lek. As swarming is the primary mating event for many bat species (Kerth & Morf, 2004; Rivers et al., 2005; Veith et al., 2004), further genetic research is needed to address swarming mating systems. The use of infrared light has also seen an increase in use and has provided valuable information on group composition and stability, and flight behaviour (Balmori, 2017; Günther et al., 2016; Rodríguez‐Herrera et al., 2019). However, the use of other emerging or increasingly accessible technology remains relatively uncommon in the study of bat mating systems. Radio or GPS telemetry could prove vital for understanding the finer intricacies of different bat mating systems. Radio telemetry revealed territoriality associated with shelter in the short‐nosed fruit bat (*Cynopterus sphinx*) (Karuppudurai & Ramesh, 2021), and telemetry could also provide insight into extra‐pair copulations, female lek visitation, or the size of areas defended by males, for example. Continued implementation of such developing technologies could provide invaluable new information in bat mating systems. Given the incredible variety in mating systems in bats, the taxon also provides a great model for the study of ecological determinants of mating systems. Variance in resource distribution has been generally acknowledged to be the cause of the existing array of mating systems, but general patterns have not been examined. Bats exhibit a wide variety of resource use patterns, and revealing the links between the mating systems and the spatiotemporal dynamics of resources will provide a greater understanding of the socioecological determinants of social organisation.

## 6 CONCLUSION

Despite the growing body of research on bats, there remains a notable dearth of studies focused on their mating systems. Mating systems, crucial for understanding social organisation and reproductive strategies, are best understood as a continuum of male reproductive skew rather than rigid categories. Our proposed framework introduces a new category termed ‘lek‐like mating system’, bridging the gap between resource defence polygyny and lek mating systems. Application of this framework to existing data reveals that lek mating systems might be more prevalent in bats than previously believed. This novel approach is poised to stimulate further investigation into bat mating systems, offering valuable insights into mating systems across species.

## AUTHOR CONTRIBUTIONS

**David Phalen:** Conceptualization (supporting); project administration (supporting); supervision (equal); validation (equal); writing – review and editing (equal). **Annabel Dorrestein:** Conceptualization (equal); data curation (lead); formal analysis (lead); investigation (lead); methodology (lead); project administration (equal); visualization (lead); writing – original draft (lead). **Justin A. Welbergen:** Conceptualization (equal); methodology (supporting); project administration (equal); supervision (lead); validation (lead); writing – review and editing (lead). **John M. Martin:** Conceptualization (supporting); project administration (supporting); supervision (equal); validation (equal); writing – review and editing (equal). **David Westcott:** Conceptualization (supporting); project administration (supporting); supervision (equal); validation (equal); writing – review and editing (equal). **Karrie Rose:** Conceptualization (supporting); project administration (supporting); supervision (equal); validation (equal); writing – review and editing (equal).

## CONFLICT OF INTEREST STATEMENT

The authors declare no conflict of interest.

## Data Availability

The data supporting this literature review are based on metadata extracted from Google Scholar and Web of Science. The metadata utilised in this study are publicly available and can be accessed through the respective repositories or databases listed in the reference section.

## APPENDIX A

### A.1

Mating system category criteria, mating system, diet, and roost of bat species of which information on mating system is available. The colour shades represent the overarching mating systems the criteria are associated with, and correspond with the colours of Figure 2. Green corresponds with promiscuity, blue with monogamy, red with defence polygyny and purple with lek mating systems (CCL, classic clustered lek; CEL, classic exploded lek; CL, classic lek; DP, defence polygyny; FDP, female defence polygyny; LL, lek‐like; Mon, monogamy; Prom, promiscuity; RDP, resource defence polygyny)

**Table:**

Species	(1) equal chance of mating	(2) pair bond between single male and single female	(3) multiple females are defended (group/range)	(4) females obtain essential resources by mating	(5.2) females select mates based on male qualities (display/resource proxy)	(5.1) males aggregate (clustered/exploded)	Mating system	References
***Artibeus cinereus***	X	X	X	V	–	–	RDP	Kunz et al. (1994) and McCracken and Wilkinson (2000)
***Artibeus jamaicensis***	X	X	X	V	–	–	RDP	Handley et al. (1991), Kunz et al. (1983), Kunz and McCracken (1996), and Morrison (1979)
***Artibeus lituratus***	X	X	X	V	–	–	RDP	Morrison (1980) and Muñoz‐Romo et al. (2008)
***Balantiopteryx plicata***	X	X	X	X	?	Clustered	Likely CCL	Bradbury and Vehrencamp (1976, 1977)
***Balionycteris maculata***	X	X	X	V	–	–	RDP	Hodgkison et al. (2003)
***Barbastella barbastellus***	X	V	–	–	–	–	MON	McWilliam (1987a) and Vaughan (1997)
*Cardioderma cor*	X	V	–	–	–	–	MON	McWilliam (1987b) and Vaughan (1976)
*Carollia perspicillata*	X	X	X	V	–	–	RDP	Fleming (1988), Heithaus and Fleming (1978), Porter (1979), and Williams (1986)
***Chaerephon pumilus***	X	X	V	?	–	–	FDP	McWilliam (1988)
***Centurio senex***	X	X	X	X	V	Clustered	CL	Rodríguez‐Herrera et al. (2020)
*Coleura afra*	X	X	V	–	–	–	FDP	Dunlop (1997), McWilliam (1987a), and Tan et al. (1997)
***Cynopterus brachyotis***	X	X	?	V	–	–	RDP	Dunlop (1997), McWilliam (1987a), and Tan et al. (1997)
***Cynopterus horsfieldii***	X	X	X	V	–	–	RDP	Tan et al. (1999)
***Cynopterus sphinx***	X	X	X	V	–	–	RDP	Bhat and Kunz (1995), Garg et al. (2018), and Karuppudurai and Ramesh (2021)
***Desmodus rotundus***	X	X	V	–	–	–	FDP	Wilkinson (1985a, 1985b, 1986)
***Ectophylla alba***	X	X	X	?	–	–	RDP	Brooke (1990) and Rodríguez‐Herrera et al. (2019)
*Epomophorus crypturus*	X	X	X	Juveniles and females were feeding nearby, so possibly gaining resources	Possibly. Appraisal but no copulation observed	V 50 m apart	CL	McCracken and Wilkinson (2000)
***Epomophorus wahlbergi***	X	X	X	X	Possibly in or closeby feeding sites. Appraisal like *H*. *monsotrosus*, but no mating observed	V Never more than 30 m apart, but aggregated in space	CL	Fenton et al. (1985) and Wickler and Seibt (1976)
***Epomops franqueti***	X	X	X	X	Possibly, females hover in front of multiple males	Exploded	CEL	Bradbury (1977a) and Kingdon (1974)
***Erophylla sezekorni***	X	X	X	X	V	V	LL	Murray and Fleming (2008)
***Eumops floridanus***	X	X	X	V	–	–	RDP	Angell and Thompson (2015), Belwood (1981), and Braun de Torrez et al. (2016, 2020)
***Hipposideros beatus***	X	V	–	–	–	–	MON	Brosset (1982)
*Hipposideros brachyotis*	X	V	–	–	–	–	MON	Bradbury (1977b)
*Hypsignathus monstrosus*	X	X	X	X	V	Clustered	CCL	
***Kerivoula papillosa***	X	V	–	–	–	–	MON	Bradbury (1977b), Medway (1969), and Rossiter et al. (2012)
*Kerivoula picta*	X	V	–	–	–	–	MON	Bradbury (1977b) and Brosset (1962)
*Lavia frons*	X	V	–	–	–	–	MON	Happold et al. (1987), Vaughan and Vaughan (1986), and Wickler and Uhrig (1969)
*Leptonycteris nivalis*	V	–	–	–	–	–	PROM	Caballero‐Martínez (2004) in Nassar et al. (2016)
***Lophostoma silvicolum***	X	X	X	V	–	–	RDP	Dechmann and Kerth (2008)
***Macrotus californicus***	X	X	V	X	V	Clustered	CCP + FDP	Berry and Brown (1995) In McCracken and Wilkinson (2000)
***Miniopterus australis***	X	X	?	?	–	–	DP	Medway (1971)
***Miniopterus minor***	X	X	X	X	?	Clustered	likely CCL	
***Mops midas***	X	X	?	?	–	–	RDP	Andriafidison et al. (2006), Bradbury (1977b), Fenton and Rautenbach (1986), Goodman (2004), Rakotonandrasana and Goodman (2007), and Verschuren (1957)
***Myotis adversus***	X	X	X	?	?	–	RDP/LL	Dwyer (1970) and Flannery (1995) IUCN – accessed October 2019
***Myotis bocagii***	X	X	V	–	–	–	FDP	Brosset (1976)
***Myotis daubentonii***	X	X	V	–	–	–	FDP	Senior et al. (2005) and van Schaik and Kerth (2017)
***Myotis lucifugus***	V	–	–	–	–	–	PROM	Thomas et al. (1979), Wai‐Ping and Fenton (1988), and Watt and Fenton (1995)
***Myotis myotis***	X	X	X	?	?	V	RDP	Audet (1990) and McCracken and Wilkinson (2000)
***Myotis nattereri***	V	–	–	–	–	–	PROM	Rivers et al. (2005)
***Myotis septentrionalis***	V	–	–	–	–	–	PROM	Burns and Broders (2015) and Jung et al. (2004)
***Myotis sodalis***	V	–	–	–	–	–	PROM	Cope and Humphrey (1977) and Thomson (1982)
***Mystacina tuberculata***	X	X	X	X	V	Clustered	CCL	Lloyd (2001), Toth et al. (2015), and Toth et al. (2018)
*Neoromicia nana*	X	X	X	X	Possibly male quality, females have a preferred male	?	likely a form of Lek	Happold and Happold (1996), LaVal and LaVal (1977), O'Shea (1980), and Van Der Merwe and Stirnemann (2009)
***Noctilio albiventris***	X	–	–	–	–	–	PROM	Barquez et al. (1999) and Fenton et al. (1993)
***Noctilio leporinus***	X	X	V	–	–	–	FDP	Barquez et al. (1999) and Brooke (1997)
***Nyctalus leisleri***	X	X	X	?	?	–	RDP	Dondini and Vergari (2009)
***Nyctalus noctula***	X	X	X	?	?	?	RDP OR LL	Mayer (1995) and Sluiter and Van Heerdt (1964)
*Nycteris arge*	X	V	–	–	–	–	MON	Bradbury and Vehrencamp (1977) and Happold et al. (1987)
*Nycteris hispida*	X	V	–	–	–	–	MON	Bradbury and Vehrencamp (1977)
*Nycteris nana*	X	V	–	–	–	–	MON	Bradbury and Vehrencamp (1977) and Grubb (1998)
***Nycticeius humeralis***	X	X	X	V	–	–	RDP	Bain and Humphrey (1986) and Watkins and Shump Jr (1981)
*Nyctophilus gouldi*	V	–	–	–	–	–	PROM	Lunney et al. (1988) and Phillips and Inwards (1985)
***Otomops martiensseni***	X	X	?	?	–	–	DP	Fenton et al. (2002) and Richardson (1995)
***Peropteryx kappleri***	X	?	?	–	–	–	FDP	Giral et al. (1991)
*Phyllostomus discolour*	X	X	X	V	–	–	RDP	Bradbury and Vehrencamp (1977)
***Phyllostomus hastatus***	X	X	V	–	–	–	FDP	Adams and Wilkinson (2020) and McCracken and Bradbury (1981)
***Pipistrellus kuhlii***	X	X	X	X	V	V	LL?	Barak and Yom‐tov (1991)
***Pipistrellus nathusii***	X	X	X	?	?	V	LL or continuum where roosts do influence mate choice a bit, and is limiting	Gerell‐Lundberg and Gerell (1994) and Toth and Parsons (2013)
***Pipistrellus pipistrellus***	X	X	X	X	V	Clustered	CCL	Jenkins et al. (1998), Nicholls and Racey (2006), and Sachteleben and von Helversen (2006)
***Pipistrellus pygmaeus***	X	X	X	X	?	V	L	Bartonička et al. (2008) and Bartoničková et al. (2016)
***Plecotus auritus***	V	–	–	–	–	–	PROM	Burland et al. (2001), Furmankiewicz (2016), Speakman and Racey (1987), and Speakman et al. (1991)
***Pteropus alecto***	X	X	V	–	–	–	FDP	Markus (2002), Hall and Richards (2000)
***Pteropus hypomelanus***	X	X	?	?	?	–	DP	McCracken and Wilkinson (2000)
*Pteropus mariannus*	X	X	?	?	?	–	DP	Pierson and Rainey (1992) and Wiles (1987)
***Pteropus poliocephalus***	X	X	X	X	V	V	LL	Eby (1991), Nelson (1965), Spencer et al. (1991), and Welbergen (2005)
*Pteropus pselaphon*	X	X	V	–	–	–	FDP	Sugita and Ueda (2013)
***Pteropus pumilus***	X	X	?	?	?	–	DP	McCracken and Wilkinson (2000)
***Pteropus rodricensis***	X	X	?	?	?	–	RDP	Carroll and Mace (1988)
*Pteropus samoensis*	X	V	–	–	–	–	MON	Banack and Grant (2003), Brooke (2001), Cox (1983), and Pierson and Rainey (1992)
***Pteropus seychellensis***	X	X	?	?	?	–	DP	Cheke and Dahl (1981)
***Pteropus tonganus***	X	X	?	?	–	–	DP	McCracken and Wilkinson (2000)
***Pteropus vampyrus***	X	X	?	?	?	–	DP	McCracken and Wilkinson (2000)
